# Octa­akis(4-amino­pyridine)-1κ^4^ *N* ^1^,2κ^4^ *N* ^1^-aqua-2κ*O*-μ-carbonato-1:2κ^3^ *O*,*O*′:*O*′′-dinickel(II) dichloride penta­hydrate

**DOI:** 10.1107/S1600536808033321

**Published:** 2008-10-18

**Authors:** Hoong-Kun Fun, A Sinthiya, Samuel Robinson Jebas, B. Ravindran Durai Nayagam, S. Alfred Cecil Raj

**Affiliations:** a X-ray Crystallography Unit, School of Physics, Universiti Sains Malaysia, 11800 USM, Penang, Malaysia; b Department of Electronics, St Josephs College, Tiruchirappalli 620 002, India; c Department of Chemistry, Popes College, Sawyerpuram 628251, Tamil Nadu, India; d Department of Physics, St Josephs College, Tiruchirappalli 620 002, India

## Abstract

In the title compound, [Ni_2_(CO_3_)(C_5_H_6_N_2_)_8_(H_2_O)]Cl_2_·5H_2_O, one of the the Ni^II^ ions is six-coordinated in a distorted octa­hedral geometry, with the equatorial plane defined by four pyridine N atoms from four amino­pyridine ligands, the axial positions being occupied by one water O and a carbonate O atom. The other Ni^II^ ion is also six-coordinated, by four other pyridine N atoms from four other amino­pyridine ligands and two carbonate O atoms to complete a distorted octa­hedral geometry. In the crystal structure, mol­ecules are linked into an infinite three-dimensional network by O—H⋯O, N—H⋯Cl, N—H⋯O, O—H⋯N, C—H⋯O, C—H⋯N and C/N—H⋯π inter­actions involving the pyridine rings.

## Related literature

For related literature on 4-amino­pyridine, see: Judge & Bever (2006 ▶); Schwid *et al.* (1997 ▶); Strupp *et al.* (2004 ▶). For bond-length data, see: Allen *et al.* (1987 ▶); Jebas *et al.* (2007 ▶).
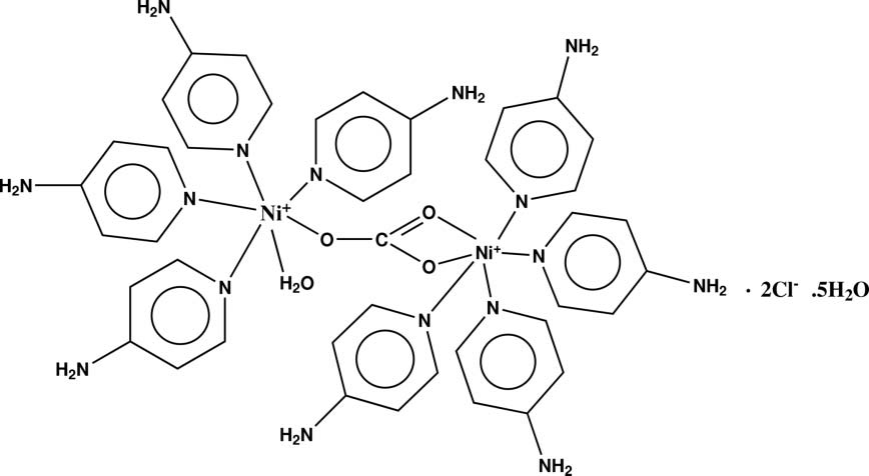


## Experimental

### 

#### Crystal data

[Ni_2_(CO_3_)(C_5_H_6_N_2_)_8_(H_2_O)]Cl_2_·5H_2_O
*M* *_r_* = 1109.37
Triclinic, 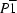

*a* = 12.8877 (3) Å
*b* = 14.7920 (3) Å
*c* = 15.0510 (3) Å
α = 82.797 (1)°
β = 68.748 (1)°
γ = 75.191 (1)°
*V* = 2583.59 (9) Å^3^
*Z* = 2
Mo *K*α radiation
μ = 0.90 mm^−1^
*T* = 100.0 (1) K
0.73 × 0.25 × 0.21 mm

#### Data collection

Bruker SMART APEXII CCD area-detector diffractometer
Absorption correction: multi-scan (*SADABS*; Bruker, 2005 ▶) *T*_min_ = 0.560, *T*_max_ = 0.834
42253 measured reflections
13659 independent reflections
10282 reflections with *I* > 2σ(*I*)
*R*_int_ = 0.046

#### Refinement

*R*[*F* ^2^ > 2σ(*F* ^2^)] = 0.065
*wR*(*F* ^2^) = 0.180
*S* = 1.04
13659 reflections
647 parameters
H-atom parameters constrained
Δρ_max_ = 2.22 e Å^−3^
Δρ_min_ = −1.94 e Å^−3^

### 

Data collection: *APEX2* (Bruker, 2005 ▶); cell refinement: *APEX2*; data reduction: *SAINT* (Bruker, 2005 ▶); program(s) used to solve structure: *SHELXTL* (Sheldrick, 2008 ▶); program(s) used to refine structure: *SHELXTL*; molecular graphics: *SHELXTL*; software used to prepare material for publication: *SHELXTL* and *PLATON* (Spek, 2003 ▶).

## Supplementary Material

Crystal structure: contains datablocks global, I. DOI: 10.1107/S1600536808033321/at2653sup1.cif

Structure factors: contains datablocks I. DOI: 10.1107/S1600536808033321/at2653Isup2.hkl

Additional supplementary materials:  crystallographic information; 3D view; checkCIF report

## Figures and Tables

**Table 1:** Hydrogen-bond geometry (Å, °)

*D*—H⋯*A*	*D*—H	H⋯*A*	*D*⋯*A*	*D*—H⋯*A*
O1*W*—H2*W*1⋯O2*W*^i^	0.85	2.04	2.807 (4)	150
N2—H2*B*⋯Cl1^ii^	0.86	2.44	3.283 (4)	166
O2*W*—H2*W*2⋯O1*W*^i^	0.85	2.35	2.807 (4)	114
N4—H4*A*⋯Cl2^iii^	0.86	2.61	3.405 (4)	153
N6—H6*A*⋯Cl1^i^	0.86	2.45	3.303 (4)	170
N8—H8*B*⋯O1^iv^	0.86	2.41	3.218 (4)	157
N8—H8*B*⋯O2^iv^	0.86	2.36	3.118 (4)	147
O5*WA*—H2*W*5⋯Cl2^i^	0.85	2.50	3.314 (6)	161
N10—H10*A*⋯O2^v^	0.86	2.10	2.880 (4)	151
N10—H10*B*⋯Cl1^ii^	0.86	2.48	3.308 (3)	162
O5*WB*—H1*WA*⋯O1*W*^i^	0.85	2.14	2.843 (7)	140
O5*WB*—H2*WB*⋯N14^vi^	0.85	2.39	3.175 (8)	154
N12—H12*A*⋯Cl2^iv^	0.86	2.74	3.401 (4)	135
N14—H14*A*⋯Cl1^vii^	0.86	2.47	3.318 (4)	168
N16—H16*B*⋯Cl2^viii^	0.86	2.54	3.364 (4)	162
C6—H6⋯N10^v^	0.93	2.49	3.352 (5)	155
C26—H26⋯N8^iv^	0.93	2.57	3.413 (5)	151
O1*W*—H1*W*1⋯O3	0.85	1.68	2.525 (3)	171
O2*W*—H1*W*2⋯Cl2	0.85	2.53	3.155 (4)	132
O2*W*—H2*W*2⋯O3*W*	0.85	2.40	2.820 (8)	111
N6—H6*B*⋯O2*W*	0.86	2.22	2.971 (5)	145
O4*W*—H1*W*4⋯Cl2	0.85	1.76	2.591 (8)	166
N8—H8*A*⋯Cl1	0.86	2.50	3.350 (3)	169
O5*WA*—H1*W*5⋯O1*W*	0.85	2.24	2.802 (6)	124
O6*WA*—H2*W*6⋯O4*W*	0.85	2.03	2.870 (7)	170
N16—H16*A*⋯Cl1	0.86	2.45	3.301 (4)	170
C1—H1⋯O3	0.93	2.43	3.020 (4)	121
C6—H6⋯O2	0.93	2.58	3.224 (4)	127
C15—H15⋯N1	0.93	2.57	3.065 (4)	114
C26—H26⋯O1	0.93	2.36	2.982 (5)	124
C15—H15⋯*Cg*1	0.93	2.86	3.559 (5)	133
C22—H22⋯*Cg*1^v^	0.93	2.95	3.764 (5)	147
N4—H4*B*⋯*Cg*2^iii^	0.86	2.84	3.668 (5)	163
C1—H1⋯*Cg*3	0.93	2.99	3.653 (5)	130
N12—H12*B*⋯*Cg*3^ix^	0.86	2.92	3.783 (5)	177

Symmetry codes: (i) 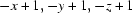; (ii) 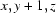; (iii) 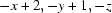; (iv) 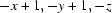; (v) 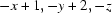; (vi) 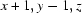; (vii) 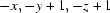; (viii) 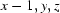; (ix) 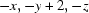. *Cg*1, *Cg*2 and *Cg*3 are the centroids of the N1/C1–C5, N7/C16–C20 and N9/C21–C25 rings, respectively.

**Fig. 1.**
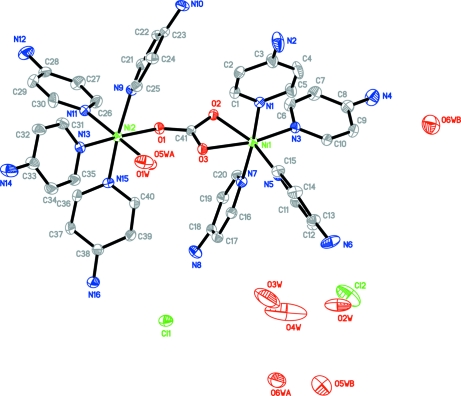
The molecular structure of the title compound, showing 50% probability displacement ellipsoids and the atom numbering scheme.

**Fig. 2.**
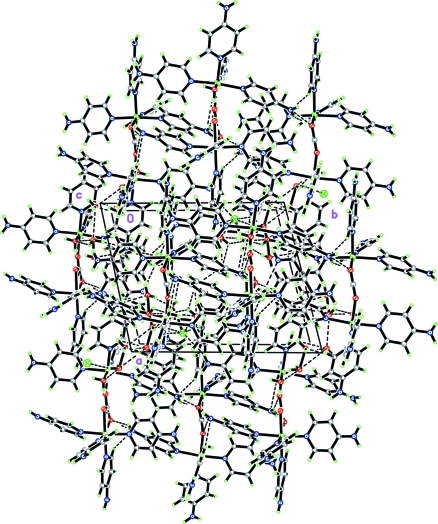
The crystal packing of the title compound, viewed along the c axis.

## supplementary crystallographic information

### Comment

4-Aminopyridine (Fampridine) is used clinically in Lambert-Eaton myasthenic syndrome and multiple sclerosis because by blocking the potassium channels it prolongs action potentials thereby increasing transmitter release at the neuromuscular junction (Judge & Bever, 2006; Schwid *et al.*,1997; Strupp *et al.*, 2004). As a part of our investigation of the binding modes of 4-aminopyridine with the metals, we report here the crystal structure of the title compound, (I).

In the asymmetric unit of the title compound, both of the Ni^II^ ions have distorted octahedral geometry. The equatorial plane in Ni1 is formed by four N pyridine atoms from four aminopyridine ligands, the axial positions being occupied by one water oxygen atom and a carbonate oxygen atom. In Ni2, the equatorial plane is formed by four other pyridine N atoms from four other aminopyridine ligands, the axial positions being occupied by two carbonate oxygen atoms. Two chlorine and five other water molecules are also present within the asymmetric unit (Fig. 1). Two of these water molecules are disordered with the fixed occupany of 0.5:0.5. The bond lengths and angles are found to have normal values (Jebas *et al.*, 2007; Allen *et al.*, 1987).

The crystal packing is consolidated by intramolecular and intermolecular O—H···O, N—H···Cl, N—H···O, O—H···N, C—H···O and C—H···N hydrogen bonds to form an infinite three dimensional network. (C/N—H···π) interactions involving the pyridine rings are also observed.

### Experimental

A solution of 4-aminopyridine (0.376 g) in methanol (20 ml) was added to a solution of NiCl_2_.6H_2_O (.237 g) in methanol (20 ml) and the mixture was stirred at a temperature of 303 K for 12 h. The clear blue solution obtained was filtered and allowed to evaporate slowly. Blue crystals of the title compound were obtained after two weeks.

### Refinement

All the hydrogen atoms were positioned geometrically [C—H=0.93 Å; N—H=0.86 Å and O—H=0.85 Å] and refined using a riding model, with *U*_iso_(H)=1.2–1.5U_equ_(C,*N* and O). The two disordered water molecules are refined with the fixed site occupancy of 0.5:0.5.

### Crystal data

**Table:**

[Ni_2_(CO_3_)(C_5_H_6_N_2_)_8_(H_2_O)]Cl_2_·5H_2_O	*Z* = 2
*M**_r_* = 1109.37	*F*(000) = 1160
Triclinic, *P*1	*D*_x_ = 1.426 Mg m^−^^3^
Hall symbol: -P 1	Mo *K*α radiation, λ = 0.71073 Å
*a* = 12.8877 (3) Å	Cell parameters from 8874 reflections
*b* = 14.7920 (3) Å	θ = 2.5–31.9°
*c* = 15.0510 (3) Å	µ = 0.90 mm^−^^1^
α = 82.797 (1)°	*T* = 100 K
β = 68.748 (1)°	Block, blue
γ = 75.191 (1)°	0.73 × 0.25 × 0.21 mm
*V* = 2583.59 (9) Å^3^	

### Data collection

**Table:**

Bruker SMART APEXII CCD area-detector diffractometer	13659 independent reflections
Radiation source: fine-focus sealed tube	10282 reflections with *I* > 2σ(*I*)
graphite	*R*_int_ = 0.046
φ and ω scans	θ_max_ = 29.0°, θ_min_ = 1.7°
Absorption correction: multi-scan (SADABS; Bruker, 2005)	*h* = −17→17
*T*_min_ = 0.560, *T*_max_ = 0.834	*k* = −20→20
42253 measured reflections	*l* = −20→20

### Refinement

**Table:**

Refinement on *F*^2^	Primary atom site location: structure-invariant direct methods
Least-squares matrix: full	Secondary atom site location: difference Fourier map
*R*[*F*^2^ > 2σ(*F*^2^)] = 0.065	Hydrogen site location: inferred from neighbouring sites
*wR*(*F*^2^) = 0.180	H-atom parameters constrained
*S* = 1.04	*w* = 1/[σ^2^(*F*_o_^2^) + (0.0775*P*)^2^ + 7.7222*P*] where *P* = (*F*_o_^2^ + 2*F*_c_^2^)/3
13659 reflections	(Δ/σ)_max_ < 0.001
647 parameters	Δρ_max_ = 2.22 e Å^−^^3^
0 restraints	Δρ_min_ = −1.94 e Å^−^^3^

### Special details

**Table:**

Experimental. The data was collected with the Oxford Cyrosystem Cobra low-temperature attachment.
Geometry. All esds (except the esd in the dihedral angle between two l.s. planes) are estimated using the full covariance matrix. The cell esds are taken into account individually in the estimation of esds in distances, angles and torsion angles; correlations between esds in cell parameters are only used when they are defined by crystal symmetry. An approximate (isotropic) treatment of cell esds is used for estimating esds involving l.s. planes.
Refinement. Refinement of *F*^2^ against ALL reflections. The weighted *R*-factor *wR* and goodness of fit *S* are based on *F*^2^, conventional *R*-factors *R* are based on *F*, with *F* set to zero for negative *F*^2^. The threshold expression of *F*^2^ > σ(*F*^2^) is used only for calculating *R*-factors(gt) *etc*. and is not relevant to the choice of reflections for refinement. *R*-factors based on *F*^2^ are statistically about twice as large as those based on *F*, and *R*- factors based on ALL data will be even larger.

### Fractional atomic coordinates and isotropic or equivalent isotropic displacement parameters (Å^2^)

**Table:**

	*x*	*y*	*z*	*U*_iso_*/*U*_eq_	Occ. (<1)
C1	0.5271 (3)	0.9042 (3)	0.2051 (3)	0.0233 (7)	
H1	0.4600	0.8895	0.2080	0.028*	
C2	0.5181 (3)	0.9898 (2)	0.2360 (3)	0.0231 (7)	
H2	0.4471	1.0308	0.2586	0.028*	
C3	0.6164 (3)	1.0154 (3)	0.2336 (3)	0.0309 (9)	
C4	0.7188 (3)	0.9500 (3)	0.1959 (4)	0.0396 (11)	
H4	0.7874	0.9639	0.1902	0.048*	
C5	0.7195 (3)	0.8659 (3)	0.1673 (3)	0.0298 (8)	
H5	0.7896	0.8238	0.1438	0.036*	
C6	0.8176 (3)	0.6901 (3)	−0.0318 (3)	0.0252 (7)	
H6	0.7548	0.7170	−0.0504	0.030*	
C7	0.9231 (3)	0.6713 (3)	−0.1012 (3)	0.0285 (8)	
H7	0.9303	0.6853	−0.1648	0.034*	
C8	1.0202 (3)	0.6312 (3)	−0.0768 (3)	0.0249 (7)	
C9	1.0021 (3)	0.6128 (3)	0.0207 (3)	0.0240 (7)	
H9	1.0635	0.5865	0.0413	0.029*	
C10	0.8929 (3)	0.6341 (2)	0.0853 (3)	0.0213 (7)	
H10	0.8833	0.6213	0.1496	0.026*	
C11	0.7216 (3)	0.5782 (2)	0.2989 (2)	0.0210 (7)	
H11	0.7480	0.5353	0.2510	0.025*	
C12	0.7460 (3)	0.5495 (3)	0.3803 (3)	0.0237 (7)	
H12	0.7865	0.4887	0.3869	0.028*	
C13	0.7097 (3)	0.6124 (3)	0.4542 (3)	0.0248 (7)	
C14	0.6474 (4)	0.7018 (3)	0.4381 (3)	0.0272 (8)	
H14	0.6208	0.7464	0.4844	0.033*	
C15	0.6256 (3)	0.7235 (3)	0.3543 (3)	0.0236 (7)	
H15	0.5830	0.7830	0.3463	0.028*	
C16	0.5630 (3)	0.5103 (2)	0.2170 (2)	0.0206 (7)	
H16	0.5448	0.5317	0.2776	0.025*	
C17	0.5325 (3)	0.4300 (3)	0.2104 (2)	0.0215 (7)	
H17	0.4979	0.3970	0.2654	0.026*	
C18	0.5538 (3)	0.3976 (2)	0.1201 (2)	0.0193 (6)	
C19	0.6132 (3)	0.4486 (3)	0.0408 (2)	0.0219 (7)	
H19	0.6325	0.4291	−0.0207	0.026*	
C20	0.6421 (3)	0.5268 (2)	0.0552 (2)	0.0208 (7)	
H20	0.6816	0.5590	0.0017	0.025*	
C21	0.2826 (3)	0.9510 (3)	0.0819 (2)	0.0215 (7)	
H21	0.2953	0.9136	0.0320	0.026*	
C22	0.3296 (3)	1.0281 (2)	0.0609 (2)	0.0218 (7)	
H22	0.3710	1.0426	−0.0018	0.026*	
C23	0.3147 (3)	1.0847 (2)	0.1346 (3)	0.0205 (7)	
C24	0.2439 (3)	1.0618 (2)	0.2266 (3)	0.0224 (7)	
H24	0.2269	1.0993	0.2775	0.027*	
C25	0.2001 (3)	0.9832 (2)	0.2403 (3)	0.0212 (7)	
H25	0.1546	0.9690	0.3017	0.025*	
C26	0.1996 (3)	0.7820 (3)	−0.0090 (3)	0.0255 (7)	
H26	0.2726	0.7480	−0.0131	0.031*	
C27	0.1732 (4)	0.7973 (3)	−0.0911 (3)	0.0306 (8)	
H27	0.2278	0.7743	−0.1483	0.037*	
C28	0.0646 (3)	0.8472 (3)	−0.0888 (3)	0.0253 (7)	
C29	−0.0111 (3)	0.8795 (3)	0.0001 (3)	0.0280 (8)	
H29	−0.0845	0.9140	0.0062	0.034*	
C30	0.0226 (3)	0.8603 (3)	0.0787 (3)	0.0278 (8)	
H30	−0.0307	0.8817	0.1371	0.033*	
C31	−0.0446 (3)	0.9253 (3)	0.3014 (3)	0.0237 (7)	
H31	−0.0175	0.9659	0.2506	0.028*	
C32	−0.1482 (3)	0.9588 (3)	0.3702 (3)	0.0281 (8)	
H32	−0.1890	1.0198	0.3651	0.034*	
C33	−0.1919 (3)	0.8998 (3)	0.4486 (3)	0.0269 (8)	
C34	−0.1233 (3)	0.8102 (3)	0.4509 (3)	0.0280 (8)	
H34	−0.1471	0.7684	0.5015	0.034*	
C35	−0.0205 (3)	0.7837 (3)	0.3785 (3)	0.0231 (7)	
H35	0.0237	0.7239	0.3827	0.028*	
C36	0.0675 (3)	0.6296 (3)	0.2273 (3)	0.0229 (7)	
H36	0.0080	0.6764	0.2184	0.028*	
C37	0.0479 (3)	0.5409 (3)	0.2534 (3)	0.0256 (8)	
H37	−0.0224	0.5291	0.2612	0.031*	
C38	0.1362 (3)	0.4684 (2)	0.2680 (2)	0.0216 (7)	
C39	0.2416 (3)	0.4919 (2)	0.2481 (2)	0.0210 (7)	
H39	0.3046	0.4457	0.2513	0.025*	
C40	0.2520 (3)	0.5823 (2)	0.2239 (2)	0.0198 (6)	
H40	0.3221	0.5960	0.2138	0.024*	
C41	0.4459 (3)	0.7377 (2)	0.1216 (2)	0.0170 (6)	
N1	0.6252 (2)	0.8396 (2)	0.1708 (2)	0.0184 (6)	
N2	0.6106 (3)	1.0977 (3)	0.2669 (4)	0.0489 (11)	
H2A	0.6719	1.1119	0.2653	0.059*	
H2B	0.5455	1.1361	0.2896	0.059*	
N3	0.7989 (2)	0.6720 (2)	0.0625 (2)	0.0191 (6)	
N4	1.1259 (3)	0.6082 (3)	−0.1450 (2)	0.0350 (8)	
H4A	1.1338	0.6183	−0.2043	0.042*	
H4B	1.1847	0.5835	−0.1286	0.042*	
N5	0.6625 (3)	0.6635 (2)	0.2825 (2)	0.0199 (6)	
N6	0.7329 (4)	0.5873 (3)	0.5356 (2)	0.0377 (9)	
H6A	0.7092	0.6268	0.5800	0.045*	
H6B	0.7713	0.5319	0.5431	0.045*	
N7	0.6177 (2)	0.5606 (2)	0.1413 (2)	0.0190 (6)	
N8	0.5179 (3)	0.3224 (2)	0.1102 (2)	0.0236 (6)	
H8A	0.4811	0.2931	0.1599	0.028*	
H8B	0.5320	0.3039	0.0542	0.028*	
N9	0.2186 (2)	0.9262 (2)	0.1713 (2)	0.0196 (6)	
N10	0.3662 (3)	1.1568 (2)	0.1190 (2)	0.0251 (6)	
H10A	0.4098	1.1691	0.0625	0.030*	
H10B	0.3555	1.1903	0.1656	0.030*	
N11	0.1267 (2)	0.8131 (2)	0.0769 (2)	0.0195 (6)	
N12	0.0350 (3)	0.8655 (3)	−0.1682 (3)	0.0366 (8)	
H12A	−0.0321	0.8979	−0.1645	0.044*	
H12B	0.0833	0.8448	−0.2222	0.044*	
N13	0.0203 (2)	0.8390 (2)	0.3017 (2)	0.0197 (6)	
N14	−0.2945 (3)	0.9289 (3)	0.5175 (3)	0.0412 (10)	
H14A	−0.3195	0.8917	0.5648	0.049*	
H14B	−0.3346	0.9846	0.5139	0.049*	
N15	0.1660 (2)	0.6529 (2)	0.2140 (2)	0.0193 (6)	
N16	0.1213 (3)	0.3815 (2)	0.2988 (3)	0.0299 (7)	
H16A	0.1770	0.3388	0.3067	0.036*	
H16B	0.0560	0.3687	0.3106	0.036*	
Ni1	0.62871 (4)	0.69875 (3)	0.15544 (3)	0.01710 (11)	
Ni2	0.18322 (4)	0.79166 (3)	0.19613 (3)	0.01683 (11)	
Cl1	0.35627 (8)	0.23887 (6)	0.31722 (6)	0.02547 (19)	
Cl2	0.90118 (15)	0.27828 (13)	0.34939 (11)	0.0716 (5)	
O1	0.3536 (2)	0.74562 (17)	0.10538 (17)	0.0188 (5)	
O1W	0.2427 (2)	0.76585 (17)	0.31497 (17)	0.0207 (5)	
H1W1	0.3100	0.7507	0.2748	0.031*	
H2W1	0.2249	0.7160	0.3444	0.031*	
O2	0.5421 (2)	0.73727 (17)	0.05345 (16)	0.0181 (5)	
O3	0.4495 (2)	0.72752 (17)	0.20747 (17)	0.0191 (5)	
O2W	0.7793 (4)	0.3796 (2)	0.5453 (2)	0.0571 (11)	
H1W2	0.8431	0.3488	0.5096	0.086*	
H2W2	0.7326	0.3440	0.5630	0.086*	
O3W	0.5677 (5)	0.4100 (4)	0.5178 (5)	0.111 (2)	
H1W3	0.5155	0.3898	0.5629	0.167*	
H2W3	0.5534	0.4106	0.4666	0.167*	
O4W	0.7123 (7)	0.2467 (3)	0.3499 (3)	0.123 (3)	
H1W4	0.7685	0.2667	0.3496	0.185*	
H2W4	0.6849	0.2803	0.3099	0.185*	
O5WA	0.1451 (6)	0.8802 (4)	0.4730 (4)	0.0363 (14)	0.50
H1W5	0.1303	0.8712	0.4246	0.054*	0.50
H2W5	0.1447	0.8301	0.5076	0.054*	0.50
O5WB	0.7622 (7)	0.1175 (5)	0.5465 (5)	0.0505 (18)	0.50
H1WA	0.7273	0.1482	0.5969	0.076*	0.50
H2WB	0.7478	0.0635	0.5579	0.076*	0.50
O6WA	0.6332 (5)	0.1318 (4)	0.5175 (4)	0.0502 (18)	0.50
H1W6	0.5629	0.1310	0.5350	0.075*	0.50
H2W6	0.6478	0.1688	0.4689	0.075*	0.50
O6WB	0.9981 (5)	0.9408 (4)	0.5318 (4)	0.0533 (18)	0.50
H1WC	1.0098	0.9362	0.5844	0.080*	0.50
H2WD	0.9477	0.9104	0.5386	0.080*	0.50

### Atomic displacement parameters (Å^2^)

**Table:**

	*U*^11^	*U*^22^	*U*^33^	*U*^12^	*U*^13^	*U*^23^
C1	0.0196 (17)	0.0245 (17)	0.0278 (18)	−0.0055 (14)	−0.0111 (14)	0.0014 (14)
C2	0.0176 (16)	0.0197 (16)	0.0283 (18)	0.0018 (13)	−0.0076 (14)	−0.0015 (14)
C3	0.0219 (18)	0.0227 (18)	0.047 (2)	−0.0037 (15)	−0.0104 (17)	−0.0039 (17)
C4	0.0177 (18)	0.030 (2)	0.072 (3)	−0.0066 (16)	−0.012 (2)	−0.011 (2)
C5	0.0179 (17)	0.0234 (18)	0.045 (2)	−0.0036 (14)	−0.0072 (16)	−0.0042 (16)
C6	0.0218 (17)	0.0298 (19)	0.0230 (17)	−0.0004 (14)	−0.0094 (14)	−0.0033 (14)
C7	0.030 (2)	0.033 (2)	0.0204 (17)	−0.0034 (16)	−0.0081 (15)	−0.0031 (15)
C8	0.0250 (18)	0.0212 (17)	0.0261 (18)	−0.0020 (14)	−0.0068 (15)	−0.0050 (14)
C9	0.0190 (16)	0.0258 (18)	0.0267 (18)	−0.0005 (14)	−0.0103 (14)	−0.0017 (14)
C10	0.0211 (17)	0.0226 (17)	0.0226 (16)	−0.0058 (13)	−0.0106 (14)	0.0014 (13)
C11	0.0230 (17)	0.0210 (16)	0.0202 (16)	−0.0067 (13)	−0.0083 (14)	0.0009 (13)
C12	0.0259 (18)	0.0212 (17)	0.0254 (17)	−0.0065 (14)	−0.0111 (15)	0.0037 (14)
C13	0.032 (2)	0.0245 (18)	0.0209 (17)	−0.0072 (15)	−0.0125 (15)	0.0017 (14)
C14	0.038 (2)	0.0227 (18)	0.0235 (17)	−0.0071 (16)	−0.0121 (16)	−0.0028 (14)
C15	0.0266 (18)	0.0218 (17)	0.0217 (17)	−0.0045 (14)	−0.0086 (14)	0.0010 (13)
C16	0.0215 (16)	0.0221 (16)	0.0202 (16)	−0.0058 (13)	−0.0092 (13)	0.0000 (13)
C17	0.0204 (16)	0.0242 (17)	0.0201 (16)	−0.0074 (14)	−0.0067 (13)	0.0027 (13)
C18	0.0156 (15)	0.0193 (16)	0.0222 (16)	−0.0029 (12)	−0.0065 (13)	−0.0007 (13)
C19	0.0229 (17)	0.0245 (17)	0.0182 (16)	−0.0047 (14)	−0.0072 (13)	−0.0017 (13)
C20	0.0212 (17)	0.0215 (16)	0.0182 (15)	−0.0040 (13)	−0.0062 (13)	0.0018 (13)
C21	0.0224 (17)	0.0237 (17)	0.0195 (16)	−0.0057 (14)	−0.0092 (14)	0.0028 (13)
C22	0.0244 (17)	0.0221 (17)	0.0195 (16)	−0.0076 (14)	−0.0085 (14)	0.0044 (13)
C23	0.0164 (15)	0.0187 (16)	0.0259 (17)	−0.0036 (12)	−0.0090 (13)	0.0057 (13)
C24	0.0248 (18)	0.0203 (16)	0.0213 (16)	−0.0052 (14)	−0.0069 (14)	−0.0005 (13)
C25	0.0195 (16)	0.0213 (16)	0.0201 (16)	−0.0037 (13)	−0.0048 (13)	0.0011 (13)
C26	0.0212 (17)	0.0283 (19)	0.0229 (17)	−0.0011 (14)	−0.0065 (14)	0.0007 (14)
C27	0.031 (2)	0.033 (2)	0.0214 (18)	−0.0005 (16)	−0.0064 (16)	−0.0007 (15)
C28	0.0305 (19)	0.0231 (17)	0.0257 (18)	−0.0097 (15)	−0.0140 (15)	0.0081 (14)
C29	0.0231 (18)	0.0297 (19)	0.033 (2)	−0.0033 (15)	−0.0147 (16)	0.0037 (15)
C30	0.0171 (17)	0.036 (2)	0.0280 (19)	−0.0014 (15)	−0.0067 (15)	−0.0048 (16)
C31	0.0238 (18)	0.0218 (17)	0.0225 (17)	−0.0061 (14)	−0.0041 (14)	0.0002 (13)
C32	0.0257 (19)	0.0174 (16)	0.033 (2)	−0.0033 (14)	−0.0025 (16)	0.0018 (14)
C33	0.0248 (18)	0.0238 (18)	0.0266 (18)	−0.0055 (15)	−0.0019 (15)	−0.0024 (14)
C34	0.0242 (18)	0.0267 (19)	0.0263 (18)	−0.0057 (15)	−0.0025 (15)	0.0042 (15)
C35	0.0222 (17)	0.0223 (17)	0.0222 (17)	−0.0043 (14)	−0.0056 (14)	0.0005 (13)
C36	0.0186 (16)	0.0242 (17)	0.0295 (18)	−0.0047 (13)	−0.0135 (14)	0.0021 (14)
C37	0.0213 (17)	0.0260 (18)	0.035 (2)	−0.0089 (14)	−0.0152 (16)	0.0023 (15)
C38	0.0250 (18)	0.0220 (17)	0.0220 (16)	−0.0085 (14)	−0.0114 (14)	0.0011 (13)
C39	0.0195 (16)	0.0200 (16)	0.0227 (16)	−0.0016 (13)	−0.0086 (14)	0.0001 (13)
C40	0.0174 (16)	0.0207 (16)	0.0219 (16)	−0.0037 (13)	−0.0076 (13)	−0.0016 (13)
C41	0.0149 (15)	0.0159 (15)	0.0191 (15)	−0.0019 (12)	−0.0059 (12)	0.0011 (12)
N1	0.0179 (13)	0.0181 (13)	0.0195 (13)	−0.0040 (11)	−0.0075 (11)	0.0010 (11)
N2	0.0255 (18)	0.0280 (19)	0.097 (4)	−0.0023 (15)	−0.022 (2)	−0.023 (2)
N3	0.0148 (13)	0.0187 (14)	0.0227 (14)	−0.0027 (11)	−0.0060 (11)	−0.0003 (11)
N4	0.0270 (17)	0.042 (2)	0.0265 (17)	0.0020 (15)	−0.0035 (14)	−0.0072 (15)
N5	0.0196 (14)	0.0217 (14)	0.0200 (14)	−0.0053 (11)	−0.0087 (11)	0.0002 (11)
N6	0.067 (3)	0.0260 (17)	0.0275 (17)	−0.0062 (17)	−0.0294 (18)	0.0026 (14)
N7	0.0192 (14)	0.0194 (14)	0.0190 (13)	−0.0041 (11)	−0.0077 (11)	0.0002 (11)
N8	0.0257 (15)	0.0251 (15)	0.0216 (14)	−0.0123 (13)	−0.0045 (12)	−0.0037 (12)
N9	0.0174 (13)	0.0190 (14)	0.0210 (14)	−0.0054 (11)	−0.0052 (11)	0.0031 (11)
N10	0.0316 (17)	0.0232 (15)	0.0239 (15)	−0.0142 (13)	−0.0099 (13)	0.0046 (12)
N11	0.0181 (14)	0.0198 (14)	0.0201 (14)	−0.0039 (11)	−0.0070 (11)	0.0016 (11)
N12	0.045 (2)	0.038 (2)	0.0289 (18)	−0.0063 (17)	−0.0200 (16)	0.0085 (15)
N13	0.0161 (13)	0.0213 (14)	0.0208 (14)	−0.0052 (11)	−0.0053 (11)	0.0003 (11)
N14	0.0302 (19)	0.0288 (18)	0.039 (2)	−0.0005 (15)	0.0126 (16)	0.0033 (15)
N15	0.0206 (14)	0.0163 (13)	0.0209 (14)	−0.0040 (11)	−0.0080 (11)	0.0019 (11)
N16	0.0323 (18)	0.0213 (15)	0.044 (2)	−0.0120 (13)	−0.0215 (16)	0.0086 (14)
Ni1	0.0158 (2)	0.0189 (2)	0.0175 (2)	−0.00417 (16)	−0.00708 (16)	0.00114 (16)
Ni2	0.0153 (2)	0.0167 (2)	0.0176 (2)	−0.00361 (16)	−0.00508 (16)	0.00077 (15)
Cl1	0.0284 (4)	0.0199 (4)	0.0233 (4)	−0.0037 (3)	−0.0047 (3)	0.0000 (3)
Cl2	0.0797 (11)	0.0871 (11)	0.0457 (7)	−0.0515 (9)	0.0068 (7)	−0.0139 (7)
O1	0.0166 (11)	0.0220 (12)	0.0186 (11)	−0.0053 (9)	−0.0062 (9)	−0.0016 (9)
O1W	0.0202 (12)	0.0235 (12)	0.0167 (11)	−0.0054 (10)	−0.0055 (9)	0.0028 (9)
O2	0.0148 (11)	0.0217 (12)	0.0177 (11)	−0.0044 (9)	−0.0064 (9)	0.0021 (9)
O3	0.0168 (11)	0.0235 (12)	0.0182 (11)	−0.0058 (9)	−0.0071 (9)	0.0013 (9)
O2W	0.096 (3)	0.0306 (17)	0.0274 (16)	−0.0150 (18)	−0.0023 (18)	0.0028 (13)
O3W	0.080 (4)	0.072 (3)	0.157 (6)	−0.045 (3)	−0.002 (4)	0.025 (4)
O4W	0.284 (9)	0.069 (3)	0.034 (2)	−0.087 (4)	−0.043 (4)	0.000 (2)
O5WA	0.058 (4)	0.035 (3)	0.021 (3)	−0.018 (3)	−0.013 (3)	0.000 (2)
O5WB	0.057 (5)	0.045 (4)	0.047 (4)	−0.015 (4)	−0.006 (4)	−0.024 (3)
O6WA	0.041 (4)	0.034 (3)	0.060 (5)	−0.006 (3)	−0.003 (3)	0.003 (3)
O6WB	0.059 (5)	0.059 (5)	0.055 (4)	−0.023 (4)	−0.028 (4)	−0.003 (4)

### Geometric parameters (Å, °)

**Table:**

C1—N1	1.346 (5)	C32—C33	1.407 (5)
C1—C2	1.366 (5)	C32—H32	0.9300
C1—H1	0.9300	C33—N14	1.355 (5)
C2—C3	1.400 (5)	C33—C34	1.398 (5)
C2—H2	0.9300	C34—C35	1.376 (5)
C3—N2	1.348 (5)	C34—H34	0.9300
C3—C4	1.392 (6)	C35—N13	1.350 (4)
C4—C5	1.365 (6)	C35—H35	0.9300
C4—H4	0.9300	C36—N15	1.339 (4)
C5—N1	1.349 (5)	C36—C37	1.380 (5)
C5—H5	0.9300	C36—H36	0.9300
C6—N3	1.354 (5)	C37—C38	1.411 (5)
C6—C7	1.367 (5)	C37—H37	0.9300
C6—H6	0.9300	C38—N16	1.347 (4)
C7—C8	1.396 (5)	C38—C39	1.406 (5)
C7—H7	0.9300	C39—C40	1.370 (5)
C8—N4	1.364 (5)	C39—H39	0.9300
C8—C9	1.402 (5)	C40—N15	1.353 (4)
C9—C10	1.372 (5)	C40—H40	0.9300
C9—H9	0.9300	C41—O1	1.273 (4)
C10—N3	1.344 (4)	C41—O2	1.291 (4)
C10—H10	0.9300	C41—O3	1.298 (4)
C11—N5	1.342 (5)	C41—Ni1	2.498 (3)
C11—C12	1.365 (5)	N1—Ni1	2.112 (3)
C11—H11	0.9300	N2—H2A	0.8600
C12—C13	1.408 (5)	N2—H2B	0.8600
C12—H12	0.9300	N3—Ni1	2.096 (3)
C13—N6	1.350 (5)	N4—H4A	0.8600
C13—C14	1.404 (5)	N4—H4B	0.8600
C14—C15	1.372 (5)	N5—Ni1	2.087 (3)
C14—H14	0.9300	N6—H6A	0.8600
C15—N5	1.354 (5)	N6—H6B	0.8600
C15—H15	0.9300	N7—Ni1	2.126 (3)
C16—N7	1.350 (4)	N8—H8A	0.8600
C16—C17	1.368 (5)	N8—H8B	0.8600
C16—H16	0.9300	N9—Ni2	2.113 (3)
C17—C18	1.407 (5)	N10—H10A	0.8600
C17—H17	0.9300	N10—H10B	0.8600
C18—N8	1.352 (4)	N11—Ni2	2.131 (3)
C18—C19	1.409 (5)	N12—H12A	0.8600
C19—C20	1.367 (5)	N12—H12B	0.8600
C19—H19	0.9300	N13—Ni2	2.129 (3)
C20—N7	1.347 (4)	N14—H14A	0.8600
C20—H20	0.9300	N14—H14B	0.8600
C21—N9	1.361 (4)	N15—Ni2	2.095 (3)
C21—C22	1.375 (5)	N16—H16A	0.8600
C21—H21	0.9300	N16—H16B	0.8600
C22—C23	1.401 (5)	Ni1—O3	2.097 (2)
C22—H22	0.9300	Ni1—O2	2.150 (2)
C23—N10	1.348 (4)	Ni2—O1	2.109 (2)
C23—C24	1.411 (5)	Ni2—O1W	2.146 (2)
C24—C25	1.379 (5)	Cl2—H1W4	1.7605
C24—H24	0.9300	O1W—H1W1	0.8500
C25—N9	1.335 (5)	O1W—H2W1	0.8502
C25—H25	0.9300	O2W—H1W2	0.8501
C26—N11	1.347 (5)	O2W—H2W2	0.8491
C26—C27	1.374 (5)	O3W—H1W3	0.8503
C26—H26	0.9300	O3W—H2W3	0.8526
C27—C28	1.396 (6)	O4W—H1W4	0.8481
C27—H27	0.9300	O4W—H2W4	0.8504
C28—N12	1.358 (5)	O5WA—H1W5	0.8501
C28—C29	1.395 (6)	O5WA—H2W5	0.8501
C29—C30	1.376 (5)	O5WB—H1WA	0.8468
C29—H29	0.9300	O5WB—H2WB	0.8478
C30—N11	1.338 (5)	O6WA—H1W6	0.8500
C30—H30	0.9300	O6WA—H2W6	0.8500
C31—N13	1.335 (5)	O6WB—H1WC	0.8498
C31—C32	1.375 (5)	O6WB—H2WD	0.8500
C31—H31	0.9300		
			
N1—C1—C2	125.3 (3)	N16—C38—C37	122.4 (3)
N1—C1—H1	117.4	C39—C38—C37	116.0 (3)
C2—C1—H1	117.4	C40—C39—C38	120.4 (3)
C1—C2—C3	119.7 (3)	C40—C39—H39	119.8
C1—C2—H2	120.2	C38—C39—H39	119.8
C3—C2—H2	120.2	N15—C40—C39	123.6 (3)
N2—C3—C4	123.1 (4)	N15—C40—H40	118.2
N2—C3—C2	121.4 (4)	C39—C40—H40	118.2
C4—C3—C2	115.5 (4)	O1—C41—O2	121.9 (3)
C5—C4—C3	120.7 (4)	O1—C41—O3	122.1 (3)
C5—C4—H4	119.6	O2—C41—O3	116.0 (3)
C3—C4—H4	119.6	O1—C41—Ni1	172.2 (2)
N1—C5—C4	124.3 (4)	O2—C41—Ni1	59.39 (16)
N1—C5—H5	117.8	O3—C41—Ni1	57.03 (16)
C4—C5—H5	117.8	C1—N1—C5	114.4 (3)
N3—C6—C7	124.0 (3)	C1—N1—Ni1	122.6 (2)
N3—C6—H6	118.0	C5—N1—Ni1	121.4 (2)
C7—C6—H6	118.0	C3—N2—H2A	120.0
C6—C7—C8	120.2 (4)	C3—N2—H2B	120.0
C6—C7—H7	119.9	H2A—N2—H2B	120.0
C8—C7—H7	119.9	C10—N3—C6	115.3 (3)
N4—C8—C7	121.4 (4)	C10—N3—Ni1	127.2 (2)
N4—C8—C9	122.3 (4)	C6—N3—Ni1	117.4 (2)
C7—C8—C9	116.3 (3)	C8—N4—H4A	120.0
C10—C9—C8	119.4 (3)	C8—N4—H4B	120.0
C10—C9—H9	120.3	H4A—N4—H4B	120.0
C8—C9—H9	120.3	C11—N5—C15	115.3 (3)
N3—C10—C9	124.7 (3)	C11—N5—Ni1	121.6 (2)
N3—C10—H10	117.6	C15—N5—Ni1	123.1 (2)
C9—C10—H10	117.6	C13—N6—H6A	120.0
N5—C11—C12	125.1 (3)	C13—N6—H6B	120.0
N5—C11—H11	117.4	H6A—N6—H6B	120.0
C12—C11—H11	117.4	C20—N7—C16	115.6 (3)
C11—C12—C13	119.6 (3)	C20—N7—Ni1	121.5 (2)
C11—C12—H12	120.2	C16—N7—Ni1	121.4 (2)
C13—C12—H12	120.2	C18—N8—H8A	120.0
N6—C13—C14	122.5 (4)	C18—N8—H8B	120.0
N6—C13—C12	121.7 (4)	H8A—N8—H8B	120.0
C14—C13—C12	115.9 (3)	C25—N9—C21	116.0 (3)
C15—C14—C13	120.1 (3)	C25—N9—Ni2	124.1 (2)
C15—C14—H14	119.9	C21—N9—Ni2	118.8 (2)
C13—C14—H14	119.9	C23—N10—H10A	120.0
N5—C15—C14	124.0 (3)	C23—N10—H10B	120.0
N5—C15—H15	118.0	H10A—N10—H10B	120.0
C14—C15—H15	118.0	C30—N11—C26	115.3 (3)
N7—C16—C17	124.2 (3)	C30—N11—Ni2	124.7 (3)
N7—C16—H16	117.9	C26—N11—Ni2	120.0 (2)
C17—C16—H16	117.9	C28—N12—H12A	120.0
C16—C17—C18	119.7 (3)	C28—N12—H12B	120.0
C16—C17—H17	120.1	H12A—N12—H12B	120.0
C18—C17—H17	120.1	C31—N13—C35	115.3 (3)
N8—C18—C17	121.7 (3)	C31—N13—Ni2	123.8 (2)
N8—C18—C19	122.1 (3)	C35—N13—Ni2	120.7 (2)
C17—C18—C19	116.2 (3)	C33—N14—H14A	120.0
C20—C19—C18	119.4 (3)	C33—N14—H14B	120.0
C20—C19—H19	120.3	H14A—N14—H14B	120.0
C18—C19—H19	120.3	C36—N15—C40	116.2 (3)
N7—C20—C19	124.7 (3)	C36—N15—Ni2	123.0 (2)
N7—C20—H20	117.6	C40—N15—Ni2	120.4 (2)
C19—C20—H20	117.6	C38—N16—H16A	120.0
N9—C21—C22	123.8 (3)	C38—N16—H16B	120.0
N9—C21—H21	118.1	H16A—N16—H16B	120.0
C22—C21—H21	118.1	N5—Ni1—N3	97.26 (11)
C21—C22—C23	119.7 (3)	N5—Ni1—O3	100.55 (10)
C21—C22—H22	120.2	N3—Ni1—O3	161.83 (11)
C23—C22—H22	120.2	N5—Ni1—N1	89.17 (11)
N10—C23—C22	122.0 (3)	N3—Ni1—N1	93.18 (11)
N10—C23—C24	121.4 (3)	O3—Ni1—N1	90.67 (10)
C22—C23—C24	116.5 (3)	N5—Ni1—N7	92.86 (11)
C25—C24—C23	119.3 (3)	N3—Ni1—N7	90.83 (11)
C25—C24—H24	120.3	O3—Ni1—N7	84.74 (10)
C23—C24—H24	120.3	N1—Ni1—N7	175.25 (11)
N9—C25—C24	124.5 (3)	N5—Ni1—O2	162.72 (10)
N9—C25—H25	117.8	N3—Ni1—O2	99.87 (10)
C24—C25—H25	117.8	O3—Ni1—O2	62.22 (9)
N11—C26—C27	124.2 (4)	N1—Ni1—O2	92.15 (10)
N11—C26—H26	117.9	N7—Ni1—O2	84.65 (10)
C27—C26—H26	117.9	N5—Ni1—C41	131.62 (11)
C26—C27—C28	120.3 (4)	N3—Ni1—C41	130.59 (11)
C26—C27—H27	119.9	O3—Ni1—C41	31.29 (10)
C28—C27—H27	119.9	N1—Ni1—C41	94.09 (11)
N12—C28—C29	121.8 (4)	N7—Ni1—C41	81.35 (11)
N12—C28—C27	122.5 (4)	O2—Ni1—C41	31.10 (10)
C29—C28—C27	115.7 (3)	N15—Ni2—O1	88.93 (10)
C30—C29—C28	120.1 (4)	N15—Ni2—N9	174.15 (11)
C30—C29—H29	120.0	O1—Ni2—N9	85.33 (10)
C28—C29—H29	120.0	N15—Ni2—N13	92.59 (11)
N11—C30—C29	124.5 (4)	O1—Ni2—N13	172.51 (10)
N11—C30—H30	117.7	N9—Ni2—N13	92.98 (11)
C29—C30—H30	117.7	N15—Ni2—N11	90.78 (11)
N13—C31—C32	125.1 (3)	O1—Ni2—N11	90.36 (10)
N13—C31—H31	117.4	N9—Ni2—N11	90.32 (11)
C32—C31—H31	117.4	N13—Ni2—N11	96.96 (11)
C31—C32—C33	119.3 (3)	N15—Ni2—O1W	87.83 (10)
C31—C32—H32	120.4	O1—Ni2—O1W	88.61 (9)
C33—C32—H32	120.4	N9—Ni2—O1W	90.96 (10)
N14—C33—C34	122.3 (3)	N13—Ni2—O1W	84.12 (10)
N14—C33—C32	121.6 (4)	N11—Ni2—O1W	178.28 (11)
C34—C33—C32	116.1 (3)	C41—O1—Ni2	129.0 (2)
C35—C34—C33	120.0 (3)	Ni2—O1W—H1W1	87.2
C35—C34—H34	120.0	Ni2—O1W—H2W1	108.0
C33—C34—H34	120.0	H1W1—O1W—H2W1	107.7
N13—C35—C34	124.1 (3)	C41—O2—Ni1	89.51 (19)
N13—C35—H35	117.9	C41—O3—Ni1	91.67 (19)
C34—C35—H35	117.9	H1W2—O2W—H2W2	107.8
N15—C36—C37	124.6 (3)	H1W3—O3W—H2W3	107.4
N15—C36—H36	117.7	H1W4—O4W—H2W4	107.8
C37—C36—H36	117.7	H1W5—O5WA—H2W5	107.7
C36—C37—C38	119.1 (3)	H1WA—O5WB—H2WB	108.2
C36—C37—H37	120.4	H1W6—O6WA—H2W6	107.7
C38—C37—H37	120.4	H1WC—O6WB—H2WD	107.7
N16—C38—C39	121.6 (3)		
			
N1—C1—C2—C3	−0.3 (6)	C6—N3—Ni1—N5	−177.3 (3)
C1—C2—C3—N2	−177.5 (4)	C10—N3—Ni1—O3	−162.3 (3)
C1—C2—C3—C4	1.9 (6)	C6—N3—Ni1—O3	14.2 (5)
N2—C3—C4—C5	177.0 (5)	C10—N3—Ni1—N1	95.8 (3)
C2—C3—C4—C5	−2.4 (7)	C6—N3—Ni1—N1	−87.8 (3)
C3—C4—C5—N1	1.3 (8)	C10—N3—Ni1—N7	−86.8 (3)
N3—C6—C7—C8	−0.2 (6)	C6—N3—Ni1—N7	89.7 (3)
C6—C7—C8—N4	177.3 (4)	C10—N3—Ni1—O2	−171.5 (3)
C6—C7—C8—C9	−0.4 (6)	C6—N3—Ni1—O2	5.0 (3)
N4—C8—C9—C10	−177.2 (4)	C10—N3—Ni1—C41	−166.1 (3)
C7—C8—C9—C10	0.4 (5)	C6—N3—Ni1—C41	10.4 (3)
C8—C9—C10—N3	0.1 (6)	C1—N1—Ni1—N5	−99.9 (3)
N5—C11—C12—C13	−1.1 (6)	C5—N1—Ni1—N5	65.0 (3)
C11—C12—C13—N6	−179.4 (4)	C1—N1—Ni1—N3	162.9 (3)
C11—C12—C13—C14	1.2 (5)	C5—N1—Ni1—N3	−32.2 (3)
N6—C13—C14—C15	−179.6 (4)	C1—N1—Ni1—O3	0.6 (3)
C12—C13—C14—C15	−0.2 (6)	C5—N1—Ni1—O3	165.5 (3)
C13—C14—C15—N5	−1.1 (6)	C1—N1—Ni1—O2	62.9 (3)
N7—C16—C17—C18	−2.8 (6)	C5—N1—Ni1—O2	−132.3 (3)
C16—C17—C18—N8	−175.2 (3)	C1—N1—Ni1—C41	31.8 (3)
C16—C17—C18—C19	4.0 (5)	C5—N1—Ni1—C41	−163.3 (3)
N8—C18—C19—C20	176.6 (3)	C20—N7—Ni1—N5	−152.6 (3)
C17—C18—C19—C20	−2.6 (5)	C16—N7—Ni1—N5	42.1 (3)
C18—C19—C20—N7	−0.2 (6)	C20—N7—Ni1—N3	−55.3 (3)
N9—C21—C22—C23	1.6 (6)	C16—N7—Ni1—N3	139.4 (3)
C21—C22—C23—N10	174.7 (3)	C20—N7—Ni1—O3	107.1 (3)
C21—C22—C23—C24	−4.5 (5)	C16—N7—Ni1—O3	−58.3 (3)
N10—C23—C24—C25	−175.0 (3)	C20—N7—Ni1—O2	44.6 (3)
C22—C23—C24—C25	4.2 (5)	C16—N7—Ni1—O2	−120.8 (3)
C23—C24—C25—N9	−0.9 (6)	C20—N7—Ni1—C41	75.7 (3)
N11—C26—C27—C28	−0.6 (6)	C16—N7—Ni1—C41	−89.6 (3)
C26—C27—C28—N12	178.8 (4)	O2—C41—Ni1—N5	179.89 (18)
C26—C27—C28—C29	0.5 (6)	O3—C41—Ni1—N5	8.0 (3)
N12—C28—C29—C30	−179.3 (4)	O2—C41—Ni1—N3	−10.4 (2)
C27—C28—C29—C30	−0.9 (6)	O3—C41—Ni1—N3	177.73 (18)
C28—C29—C30—N11	1.5 (6)	O2—C41—Ni1—O3	171.9 (3)
N13—C31—C32—C33	0.4 (6)	O2—C41—Ni1—N1	87.37 (19)
C31—C32—C33—N14	−178.9 (4)	O3—C41—Ni1—N1	−84.53 (19)
C31—C32—C33—C34	1.3 (6)	O2—C41—Ni1—N7	−93.98 (19)
N14—C33—C34—C35	179.1 (4)	O3—C41—Ni1—N7	94.1 (2)
C32—C33—C34—C35	−1.0 (6)	O3—C41—Ni1—O2	−171.9 (3)
C33—C34—C35—N13	−1.0 (6)	C36—N15—Ni2—O1	−147.6 (3)
N15—C36—C37—C38	0.4 (6)	C40—N15—Ni2—O1	40.4 (3)
C36—C37—C38—N16	176.1 (4)	C36—N15—Ni2—N13	39.8 (3)
C36—C37—C38—C39	−4.2 (5)	C40—N15—Ni2—N13	−132.2 (3)
N16—C38—C39—C40	−175.0 (3)	C36—N15—Ni2—N11	−57.2 (3)
C37—C38—C39—C40	5.3 (5)	C40—N15—Ni2—N11	130.8 (3)
C38—C39—C40—N15	−2.7 (6)	C36—N15—Ni2—O1W	123.8 (3)
C2—C1—N1—C5	−0.9 (5)	C40—N15—Ni2—O1W	−48.2 (3)
C2—C1—N1—Ni1	165.0 (3)	C25—N9—Ni2—O1	−127.7 (3)
C4—C5—N1—C1	0.4 (6)	C21—N9—Ni2—O1	40.0 (3)
C4—C5—N1—Ni1	−165.7 (4)	C25—N9—Ni2—N13	45.0 (3)
C9—C10—N3—C6	−0.7 (5)	C21—N9—Ni2—N13	−147.3 (3)
C9—C10—N3—Ni1	175.8 (3)	C25—N9—Ni2—N11	142.0 (3)
C7—C6—N3—C10	0.7 (6)	C21—N9—Ni2—N11	−50.4 (3)
C7—C6—N3—Ni1	−176.2 (3)	C25—N9—Ni2—O1W	−39.1 (3)
C12—C11—N5—C15	−0.2 (5)	C21—N9—Ni2—O1W	128.5 (3)
C12—C11—N5—Ni1	−179.2 (3)	C31—N13—Ni2—N15	−151.3 (3)
C14—C15—N5—C11	1.3 (5)	C35—N13—Ni2—N15	33.6 (3)
C14—C15—N5—Ni1	−179.7 (3)	C31—N13—Ni2—N9	30.5 (3)
C19—C20—N7—C16	1.6 (5)	C35—N13—Ni2—N9	−144.6 (3)
C19—C20—N7—Ni1	−164.6 (3)	C31—N13—Ni2—N11	−60.2 (3)
C17—C16—N7—C20	−0.1 (5)	C35—N13—Ni2—N11	124.7 (3)
C17—C16—N7—Ni1	166.1 (3)	C31—N13—Ni2—O1W	121.1 (3)
C24—C25—N9—C21	−2.0 (5)	C35—N13—Ni2—O1W	−54.0 (3)
C24—C25—N9—Ni2	165.9 (3)	C30—N11—Ni2—N15	104.0 (3)
C22—C21—N9—C25	1.7 (5)	C26—N11—Ni2—N15	−79.3 (3)
C22—C21—N9—Ni2	−166.9 (3)	C30—N11—Ni2—O1	−167.0 (3)
C29—C30—N11—C26	−1.5 (6)	C26—N11—Ni2—O1	9.7 (3)
C29—C30—N11—Ni2	175.4 (3)	C30—N11—Ni2—N9	−81.7 (3)
C27—C26—N11—C30	1.0 (6)	C26—N11—Ni2—N9	95.0 (3)
C27—C26—N11—Ni2	−176.0 (3)	C30—N11—Ni2—N13	11.3 (3)
C32—C31—N13—C35	−2.3 (6)	C26—N11—Ni2—N13	−172.0 (3)
C32—C31—N13—Ni2	−177.7 (3)	O2—C41—O1—Ni2	−157.8 (2)
C34—C35—N13—C31	2.6 (5)	O3—C41—O1—Ni2	23.7 (5)
C34—C35—N13—Ni2	178.1 (3)	N15—Ni2—O1—C41	−106.2 (3)
C37—C36—N15—C40	2.5 (5)	N9—Ni2—O1—C41	72.7 (3)
C37—C36—N15—Ni2	−169.8 (3)	N11—Ni2—O1—C41	163.0 (3)
C39—C40—N15—C36	−1.3 (5)	O1W—Ni2—O1—C41	−18.3 (3)
C39—C40—N15—Ni2	171.2 (3)	O1—C41—O2—Ni1	−171.0 (3)
C11—N5—Ni1—N3	−58.4 (3)	O3—C41—O2—Ni1	7.6 (3)
C15—N5—Ni1—N3	122.7 (3)	N5—Ni1—O2—C41	−0.3 (4)
C11—N5—Ni1—O3	118.0 (3)	N3—Ni1—O2—C41	172.03 (19)
C15—N5—Ni1—O3	−61.0 (3)	O3—Ni1—O2—C41	−4.74 (18)
C11—N5—Ni1—N1	−151.5 (3)	N1—Ni1—O2—C41	−94.4 (2)
C15—N5—Ni1—N1	29.6 (3)	N7—Ni1—O2—C41	82.1 (2)
C11—N5—Ni1—N7	32.8 (3)	O1—C41—O3—Ni1	170.8 (3)
C15—N5—Ni1—N7	−146.1 (3)	O2—C41—O3—Ni1	−7.8 (3)
C11—N5—Ni1—O2	114.0 (4)	N5—Ni1—O3—C41	−173.93 (19)
C15—N5—Ni1—O2	−65.0 (5)	N3—Ni1—O3—C41	−5.5 (4)
C11—N5—Ni1—C41	113.8 (3)	N1—Ni1—O3—C41	96.8 (2)
C15—N5—Ni1—C41	−65.2 (3)	N7—Ni1—O3—C41	−82.0 (2)
C10—N3—Ni1—N5	6.2 (3)	O2—Ni1—O3—C41	4.72 (18)

### Hydrogen-bond geometry (Å, °)

**Table:**

*D*—H···*A*	*D*—H	H···*A*	*D*···*A*	*D*—H···*A*
O1W—H2W1···O2W^i^	0.85	2.04	2.807 (4)	150
N2—H2B···Cl1^ii^	0.86	2.44	3.283 (4)	166
O2W—H2W2···O1W^i^	0.85	2.35	2.807 (4)	114
N4—H4A···Cl2^iii^	0.86	2.61	3.405 (4)	153
N6—H6A···Cl1^i^	0.86	2.45	3.303 (4)	170
N8—H8B···O1^iv^	0.86	2.41	3.218 (4)	157
N8—H8B···O2^iv^	0.86	2.36	3.118 (4)	147
O5WA—H2W5···Cl2^i^	0.85	2.50	3.314 (6)	161
N10—H10A···O2^v^	0.86	2.10	2.880 (4)	151
N10—H10B···Cl1^ii^	0.86	2.48	3.308 (3)	162
O5WB—H1WA···O1W^i^	0.85	2.14	2.843 (7)	140
O5WB—H2WB···N14^vi^	0.85	2.39	3.175 (8)	154
N12—H12B···Cl2^iv^	0.86	2.73	3.401 (4)	137
N14—H14A···Cl1^vii^	0.86	2.47	3.318 (4)	168
N16—H16B···Cl2^viii^	0.86	2.54	3.364 (4)	162
C6—H6···N10^v^	0.93	2.49	3.352 (5)	155
C26—H26···N8^iv^	0.93	2.57	3.413 (5)	151
O1W—H1W1···O3	0.85	1.68	2.525 (3)	171
O2W—H1W2···Cl2	0.85	2.53	3.155 (4)	132
O2W—H2W2···O3W	0.85	2.40	2.820 (8)	111
N6—H6B···O2W	0.86	2.22	2.971 (5)	145
O4W—H1W4···Cl2	0.85	1.76	2.591 (8)	166
N8—H8A···Cl1	0.86	2.50	3.350 (3)	169
O5WA—H1W5···O1W	0.85	2.24	2.802 (6)	124
O6WA—H2W6···O4W	0.85	2.03	2.870 (7)	170
N16—H16A···Cl1	0.86	2.45	3.301 (4)	170
C1—H1···O3	0.93	2.43	3.020 (4)	121
C6—H6···O2	0.93	2.58	3.224 (4)	127
C15—H15···N1	0.93	2.57	3.065 (4)	114
C26—H26···O1	0.93	2.36	2.982 (4)	124
C15—H15···Cg1	0.93	2.86	3.559 (5)	133
C22—H22···Cg1^v^	0.93	2.95	3.764 (5)	147
N4—H4B···Cg2^iii^	0.86	2.84	3.668 (5)	163
C1—H1···Cg3	0.93	2.99	3.653 (5)	130
N12—H12B···Cg3^ix^	0.86	2.92	3.783 (5)	177

Symmetry codes: (i) −*x*+1, −*y*+1, −*z*+1; (ii) *x*, *y*+1, *z*; (iii) −*x*+2, −*y*+1, −*z*; (iv) −*x*+1, −*y*+1, −*z*; (v) −*x*+1, −*y*+2, −*z*; (vi) *x*+1, *y*−1, *z*; (vii) −*x*, −*y*+1, −*z*+1; (viii) *x*−1, *y*, *z*;  (ix) −*x*, −*y*+2, −*z*.
